# Phenotypic and functional analysis of γδ T cells in the pathogenesis of human T-cell lymphotropic virus type 1 infection

**DOI:** 10.3389/fimmu.2022.920888

**Published:** 2022-08-11

**Authors:** Matias Ruggieri, Nicolás Ducasa, Claudia Juraske, Virginia Gonzalez Polo, Carolina Berini, Maria Florencia Quiroga, Petros Christopoulos, Susana Minguet, Mirna Biglione, Wolfgang W. Schamel

**Affiliations:** ^1^ Department of Immunology, Faculty of Biology, Albert-Ludwigs-University of Freiburg, Freiburg, Germany; ^2^ Signalling Research Centres Centre for Biological Signalling Studies (BIOSS) and Centre for Integrative Biological Signalling Studies (CIBSS), University of Freiburg, Freiburg, Germany; ^3^ Center of Chronic Immunodeficiency (CCI), University Clinics and Medical Faculty, Freiburg, Germany; ^4^ Institute for Clinical Pathology, University Hospital Freiburg, Freiburg, Germany; ^5^ National Scientific and Technical Research Council (CONICET), University of Buenos Aires, Institute for Biomedical Research in Retroviruses and AIDS (INBIRS), Buenos Aires, Argentina; ^6^ Spemann Graduate School of Biology and Medicine (SGBM), Freiburg, Germany; ^7^ Department of Thoracic Oncology, Thoracic Clinic at Heidelberg University Hospital, Heidelberg, Germany; ^8^ Translational Lung Research Center Heidelberg (TLRC-H), German Center for Lung Research (DZL), Heidelberg, Germany

**Keywords:** γδ T cells, HTLV-1, IFN-γ, mevalonate pathway, cytotoxicity

## Abstract

The human T-cell leukemia virus type 1 (HTLV-1) is the cause of serious malignant and inflammatory diseases, including adult T-cell leukemia and lymphoma and tropical spastic paraparesis. The potential protective role of γδ T cells in HTLV-1 infection remains unclear. Here, demonstrate that there is a decrease in the amount of Vγ9Vδ2 T cells in patients with HTLV-1, especially in those with HTLV-1 associated pathologies. This suggests that γδ T cells could be involved in controlling the virus. Indeed, we found that Vγ9Vδ2 T cells, expanded from non-infected individuals, can kill cells expressing the viral proteins HBZ and Tax and this phenotype is reversed in the presence of mevastatin. Cytotoxicity by Vγ9Vδ2 T cells was not associated with an increase of INF-γ production. In sharp contrast, killing by NK cells was reduced by Tax expression. Thus, our study provides initial evidence for a potential protective role of Vγ9Vδ2 T cells against HTLV-1 infection. Therapeutic exploitation of these insights is feasible with current technologies of T-cell therapies and could provide novel tools to prevent and treat HTLV-1-associated malignancies and neurologic complications.

## Introduction

The human T-cell leukemia virus type 1 (HTLV-1) is a retrovirus that infects at least 10 million people worldwide (1). HTLV-1 is the etiologic agent of two main diseases: a malignant neoplastic disease named adult T-cell leukemia/lymphoma (ATLL) and an inflammatory disorder, HTLV-1-associated myelopathy/tropical spastic paraparesis (HAM/TSP). Individuals with a higher proviral load (PVL) are at greater risk of developing either ATL or HAM/TSP (2). Nonetheless, about 2-5% of HTLV-1 infected individuals develop ATLL or HAM/TSP, suggesting a role of host genetic risk factors (3, 4).

Of all known proteins encoded by HTLV-1, the most studied ones are Tax and HTLV-1 basic leucine zipper factor (HBZ). Both participate in viral replication, proliferation of infected cells, propagation of the virus and have pleiotropic functions implicated in viral pathogenesis. Tax is important for initiating lymphocyte immortalization, whereas HBZ is essential for maintaining the immortalized phenotype. Both proteins change the transcription of the host cells and play a critical role in HTLV-1 infection and pathogenesis (5–7).

The main target of HTLV-1 are CD4 T cells. After infection, the virus induces an important immune dysregulation by changing the CD4 T cell immunophenotype to effector/memory T cells (8, 9). Furthermore, the infection introduces significant dysregulation of the host’s immune system at the cytokine and chemokine levels (10, 11). Additionally, the virus has also been found in other cell types, including CD8 T cells, B lymphocytes, dendritic cells, monocytes, and endothelial cells (12).

The efficacy of the immune response against HTLV-1, which involves antibody secretion and CD8 cytotoxic T cell (CTL) activation, is a fundamental factor in the outcome of diseases associated with HTLV-1 and these host components have been extensively studied (13, 14). In addition to CD8 CTLs, there are other cell populations that have cytolytic activity against virus-infected cells, such as natural killer (NK) cells and γδ T cells; however, there is little information about their specific role in HTLV-1 infection.

NK cells participate in the immune innate response against viruses and tumors, in which they exert cytolytic activity and produce various proinflammatory cytokines and chemokines (15, 16). In HTLV-1 infection, a dysregulation of NK cells is also observed. The frequency of NK cells is decreased in HAM/TSP patients and an inverse correlation was described between the PVL and the frequency of NK cells in HTLV-1 carriers (17). Nonetheless, other studies suggested that NK cells could reduce HTLV-1 PVL (18).

γδ T cells bridge the innate and adaptive immune responses. They are an important unconventional T cell subset, as they have the ability to recognize a broad range of antigens without the presence of major histocompatibility complex (MHC) molecules. γδ T cell responses are mainly induced upon the recognition of stress antigens by their γδ T cell receptors (TCRs) (19).

There are two major subsets of human γδ T cells identified by their Vδ chain. Vδ1 T cells are predominant in mucosa and epithelium, while Vδ2 T cells constitute the majority of blood γδ T cells. A third population is formed by Vδ3 T cells, which are infrequent in the peripheral blood but rich in the liver. Finally, there are human γδ T cells expressing Vδ4, Vδ5 and Vδ6, from which little is known (20, 21). The different TCRδ chains and TCRγ chains combine to form different γδ TCRs and subsequently different cell types. For example, Vδ2 mainly associates with the Vγ9 chain in cells referred to as Vγ9Vδ2 T cells representing the majority of γδ T cells in peripheral blood (22, 23). Vγ9Vδ2 TCRs react to phosphoantigens (pAgs), which are intermediates in the biosynthesis of isoprenoids and present in all host cells, albeit at low levels. Vγ9Vδ2 T cells show roughly 1000-fold higher sensitivity for microbial pAgs, such as 4-hydroxy-3-methyl-but-2-enyl pyrophosphate (HMBPP), than for vertebrate pAgs, like isopentenyl pyrophosphate (IPP). In tumor cells pAgs are often upregulated, thus also becoming targets for Vγ9Vδ2 T cells. Butyrophilin (BTN) proteins (3A1 and 2A1) are the key mediators of pAgs sensing by human Vγ9Vδ2 T cells, although the exact molecular mechanism of this recognition remains elusive (24).

Certain infections can cause immune alterations by modify the proportion of the different γδ T cells subsets. For example, a decrease of Vδ2 and an increase in Vδ1 cells has been shown in HIV-infected patients, compared to non-infected (NI) individuals, resulting in the inversion of the normal ratio of Vδ2 to Vδ1 T cells. Antiretroviral therapy failed to correct this inversion of the ratio (25). Finally, there are many reports showing that γδ T cells are involved in the control of HIV infection as well as Epstein–Barr Virus, Hepatitis B Virus and Hepatitis C Virus (26). However, little is known about the role of γδ T cells during HTLV-1 infection.

For these reasons, our main goal was to study γδ T cells to contribute to the understanding of immune dysregulations in the pathogenesis of HTLV-1 infection.

## Results

### Vγ9Vδ2 T cells are reduced in HTLV-1 patients

**Figure 1:**
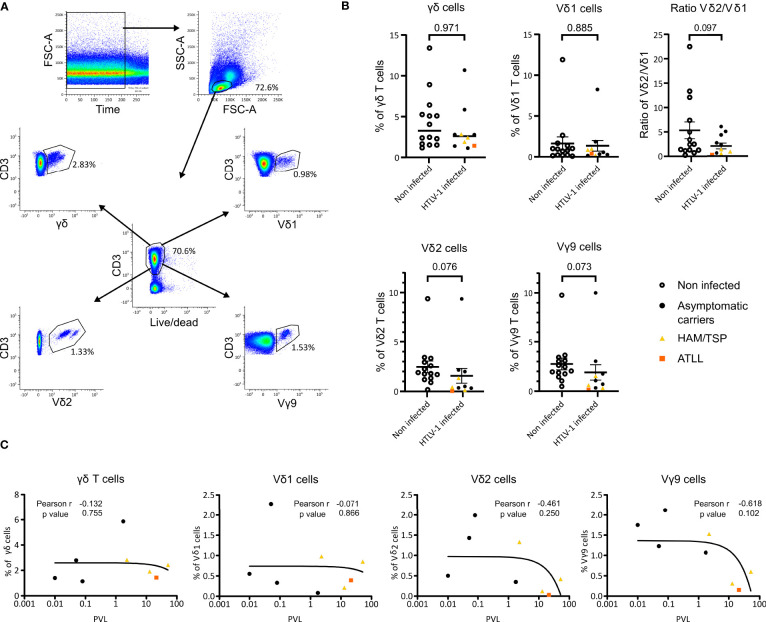
Flow cytometry analysis of γδ T cell populations in HTLV-1 patients and PVL correlation in HTLV-1 patients. **(A)** Gating strategy example. PBMCs of a non-infected individual were stained with Fixable Near-IR Dead Cell Stain, anti-CD3, anti-TCR Vγ9, anti-TCR Vδ2, anti-TCRγδ and anti-TCR Vδ1 and measured by flow cytometry. **(B)** Percentages of the γδ subsets present in NI individuals (n=14) (empty circle) and HTLV‐1‐infected patient samples clasified as AC (n=8) (black circle), HAM/TSP (n=3) (yellow triangle) and ATLL (n=1) (orange square). p-values were determined by Two-tailed, Mann Whitney test analysis. **(C)** Correlation of γδ, Vδ1, Vδ2 and Vγ9 cells with proviral load (PVL) from 4 AC (black), 3 HAM/TSP (yellow) and 1 ATLL (orange) samples was analyzed Pearson’s rank correlation coefficient by two tails.

We investigated whether HTLV-1 infection, HAM/TSP or ATLL disease would induce changes in the frequency of γδ T cells in peripheral blood T cells. We studied 24 samples corresponding to 14 NI individuals and 10 HTLV‐1‐infected samples (6 asymptomatic carriers (AC), 3 HAM/TSP and 1 ATLL samples) by flow cytometry (**Supplemental Table 1** and **Figures 1A**, **B**). **Figure 1A** shows the gating strategy used to analyze γδ T cell populations in peripheral blood.

**Figure 2:**
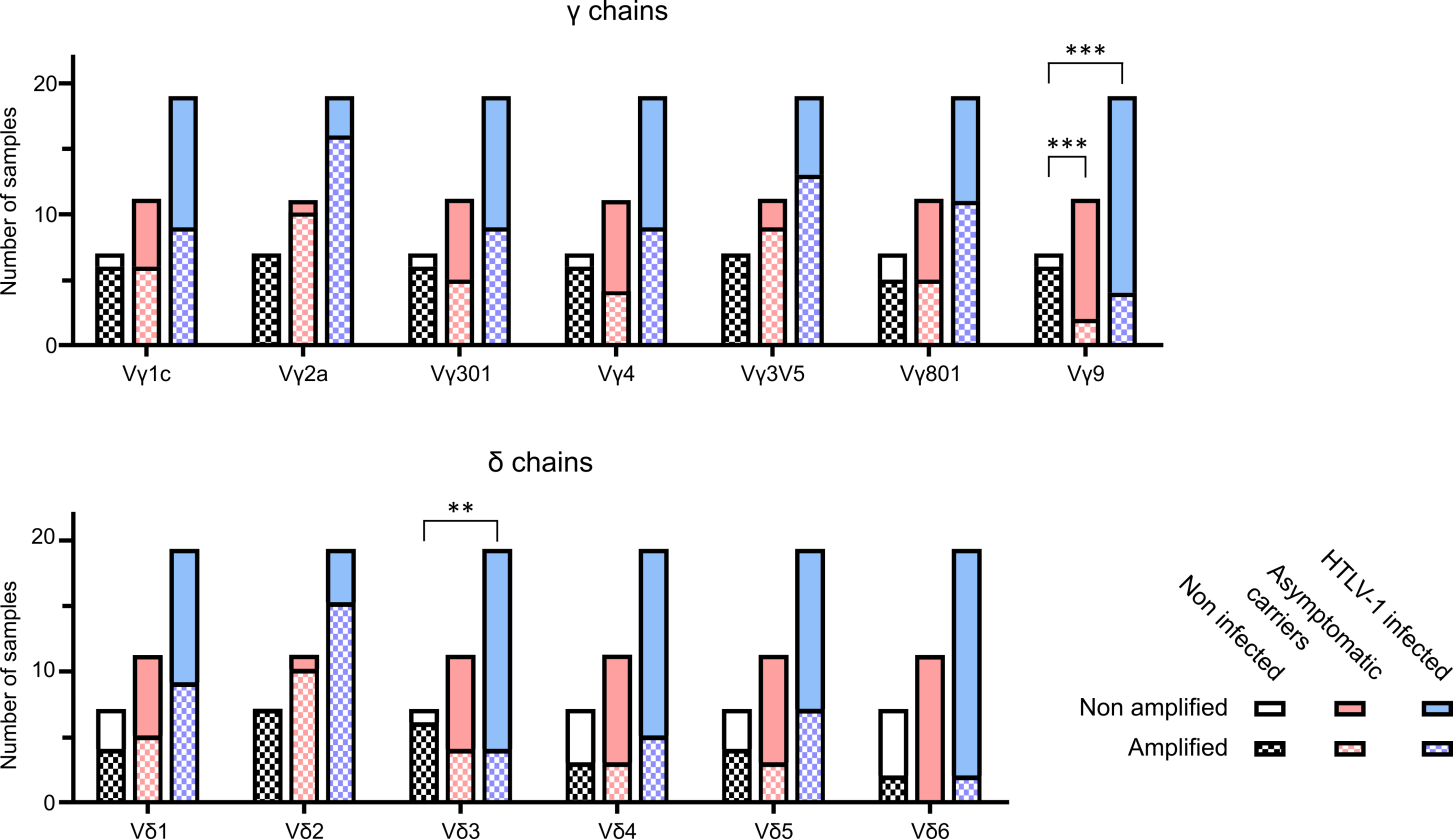
Spectratyping of γδ TCR chains. TCRγ and TCRδ spectratyping was performed with cDNA from PBMCs of 7 NI individuals, 11 AC patient samples and 8 samples with pathologies (5 HAM/TSP and 3 ATLL). The bars represent the total amonut of samples analysed, clasified as NI (black), AC (pink) and HTLV-1 infected (AC and pathologies) (light blue). Each bar contains the samples that could be amplified (square patterned bars) and the ones that could not (plain bars). The particular V regions amplified were VγI subgroup (genes Vγ1-8) (panTRGVI) and its individual functional genes Vγ3 (TRGV3), Vγ4 (TRGV5), Vγ5 (TRGV3V5), Vγ8 (TRGV8) and the single gene of the VγII subgroup Vγ9 (TRVG9), as well as the Vδ1-6 (TRDV1-6). All data was generated in 8 independent experiments. **: p<0.005; ***: p<0.001. p-values were determined by two-sided Chi-square test.

We observed a tendency for the reduction in the proportion of the Vδ2 and Vγ9 subset cells in HTLV-1-infected individuals compared to NI (p=0.076 and p=0.073, respectively) (**Figure 1B**). This tendency was confirmed by spectrotyping with a statistical significance of p<0.005 for the analysis shown in **Figure 2** and p<0.01 for the analysis in **Supplemental Table 3**. Moreover, HAM/TSP infected individuals showed a significantly lower proportion of Vδ2 and Vγ9 T cells compared to NI individuals (p=0.041 and p=0.039 respectively). We were unable to statistically assess the relevance of ATLL disease over the proportion of γδ T cells, but we could observe that the only patient included presented a very low proportion of both Vδ2 and Vγ9 T cells (**Figure 1B**, orange square). γδ and Vδ1 T cell pro-portions were similar between HTLV-1 infected and NI individuals, and HAM/TSP and ATLL individuals showed a tendency of lower γδ and Vδ1 proportions. Finally, considering only the AC samples, the p values for Vδ2 and Vγ9 are non-significant but a clear diminishing of these subsets can be observed compared to NI individuals (**Figure 1B**).

The PVL values of 4 AC, 3 HAM/TSP and 1 ATLL samples were compared with the γδ T cell frequencies, including each analyzed subset. At higher PVL, there is a tendency that the percentages of Vδ2 and Vγ9 positive cells is lower (**Figure 1C**). This effect is more pronounced in samples corresponding to the pathologies of HAM/TSP and ATLL. The decrease in total γδ T cells and in the Vδ1 subset is less pronounced, but also emphasized with the pathologies (**Figure 1C**).

The flow cytometry technique is limited by the fact that antibodies against the less frequently expressed V regions are not commercially available. Therefore, we employed TCRγδ spectratyping using a previously validated primer set that covers all expressed Vγ and Vδ genes. The spectratypes of individual patients are shown in the **Supplemental Figure 1**. A description of the clinical status is given in **Supplemental Table 2**.

We analyzed the amplification results using two different procedures. **Figure 2** shows the number of samples with detectable amplification of the different Vγ and Vδ chains. In samples of patients infected with HTLV-1 (AC and with pathologies), several Vγ and Vδ chains were less often detected compared to NI individuals. In particular, while 100% of the samples (7/7) from NI individuals showed detectable amplification of Vδ2 chains, amplification was only detected in 74% (14/19) of patients infected with HTLV-1 (AC and with pathologies)and in 90% (10/11) of the asymptomatic carriers. On the other hand, Vγ9 amplification was detectable in 86% (6/7) NI samples, but only in 18% (2/11) of the asymptomatic carriers and in 21% (4/19) of patients infected with HTLV-1 (AC and with pathologies). A chi square test showed a significant difference in the amplification of Vδ3 when comparing NI and HTLV-1 infected samples (p=0.005) and in Vγ9 when comparing NI with AC (p=0.0005) or NI with HTLV-1 infected (including pathologies) (p=0.0007).

Taken together, the results from flow cytometry and spectratyping suggest that HTLV-1 infection induces changes in the γδ T cell repertoire in peripheral blood, such as less Vγ9. In addition, Vδ3 T cells might also be reduced.

Sectratyping is a semiquantitative method that allows for the detection of clonal expansions along with diversity, denoted by the number of peaks in each family. We also used the area under each spectratyping curve as a proxy for the relative abundance of the respective γδ T cell subsets in all samples (20). The computational analysis on these data was performed based on the samples being derived from 4 categories: NI (7), AC (12), HAM/TSP (4) and ATLL (3). Except for Vδ1 and Vδ6, all γδ subsets seemed to be impaired in HTLV-1 samples concerning diversity (number of peaks in each family), number of detectable clonal expansions and relative abundance (**Supplemental Table 3**).

These findings corroborate the flow cytometry results, that symptomatic HTLV-1 patients have less γδ T cells, such as less Vγ9Vδ2 cells.

### Cells expressing HBZ and Tax proteins are more susceptible to cytolysis by Vγ9Vδ2 clones

**Figure 3:**
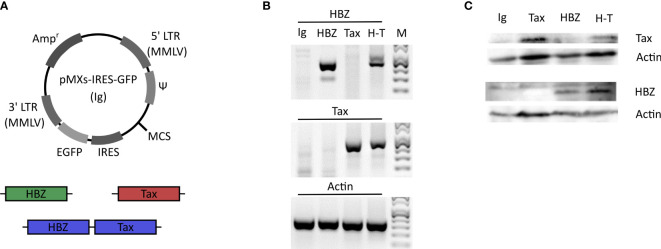
Generation of the HBZ- and Tax-expressing β2M FO-1 transductants. **(A)** Schematic diagram of the retroviral vector pMXs-IRES-GFP (Ig). HBZ (green) and Tax (red) genes or a combination of both (H-T) (blue) were introduced into the multiple cloning site (MCS). The vector contains the ampicillin-resistance gene (Amp^r^), moloney murine leukemia virus (MMLV) long terminal repeat (LTR) regions (5’LTR MMLV and 3’LTR MMLV), viral package signal (Ψ), enhanced green florescent protein (EGFP) and the internal ribosome entry site (IRES). **(B)** RT-PCR of the β2M FO-1 transductants to confirm the mRNA expresion of HBZ and Tax. Actin was used as a control. **(C)** Western blot of the β2M FO-1 transductnats to analyse the protein expression levels of HBZ and Tax in these cells. Actin was used as a loading control.

Since γδ T cells play a role in immune reactions against virus infection, we wanted to test whether Vγ9Vδ2 cells can killing cells expressing HTLV-1 proteins, HBZ or Tax. Clones of the Vγ9Vδ2 effector cells were expanded from human peripheral blood of healthy donors. As target cells we selected the human γδ T cell line Peer and the melanoma cell line β2M FO-1 and lentivirally transduced them with an empty vector encoding for GFP (Ig) or vectors encoding for HBZ and GFP, or Tax and GFP, or both and GFP (H-T) (**Figure 3A**). After transduction, cells were sorted by GFP and mRNA expression of the β2M FO-1 transductants was confirmed by RT-PCR (**Figure 3B**). Protein expression was confirmed by Western blot (**Figures 3C**). Peer transductants were successfully done for Tax and H-T (**Supplemental Figure 2**), the HBZ transductant did not express HBZ.

**Figure 4:**
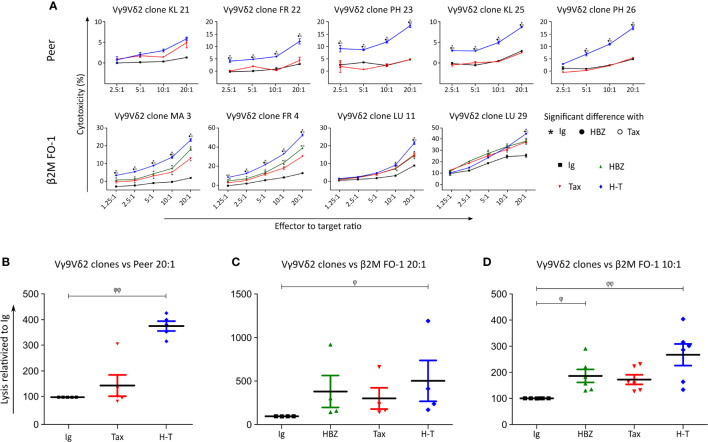
Vγ9Vδ2 clones kill Tax- and HBZ-expressing target cells. **(A)** Cytolysis of Peer cells transduced with Ig (empty vector, black), Tax (red) or H-T (blue) by five Vγ9Vδ2 T clones from three different donors (KL, FR, PH) and cytolysis of β2M FO-1 cells transduced with Ig (control), HBZ, Tax and H-T by four Vγ9Vδ2 T clones from three different donors (MA, FR, LU) was determined with the Cr^51^ release assay. The statistical analysis was performed for each clone individually, based on the technical triplicates. The *p*-values were determined by Two-way ANOVA, with Bonferroni post-test and significance is indicated when p<0.001. **(B, C)** Percentage of increased cytolysis in a dilution 1/20 effector to target cells (Peer and β2M FO-1), for all Vγ9Vδ2 clones used in one experiment **(A)** (n=5 and n=4, respectively). **(D)** Percentage of increased cytolysis in a dilution 1/10 effector to target cells, for all Vγ9Vδ2 clones used in **(A)** (β2M FO-1) plus clones MK (2, 6, 7) (n=7); two independent experiments. φ: p<0.05; φ φ: p<0.01; p-values were determined by Kruskal-Wallis test, with Dunns post-test.

Next, a killing assay based on Cr51 release by the target cells was carried out by co-culturing of the effector Vγ9Vδ2 T clones with the target cells (**Figure 4**). Peer cytolysis by all five Vγ9Vδ2 T clones from three different donors was in-creased when Peer co-expressed HBZ and Tax (H-T). No difference was observed when Tax was expressed alone com-pared to control (Ig). β2M FO-1 cells expressing HBZ, Tax or H-T underwent higher cytolysis upon incubation with four Vγ9Vδ2 clones from three different donors compared to the control (**Figure 4A**).

Pooling all Vγ9Vδ2 clones for a statistical analysis indicated a significant increase in susceptibility of Peer and β2M FO-1 cells simultaneously expressing HBZ and Tax (H-T, **Figures 4B**, **C**). In addition, the expression of HBZ alone significantly increased the cytolysis of β2M FO-1 cells by the Vγ9Vδ2 clones and a tendency of the same effect is observed for Tax (**Figures 4C**, **D**). These results were reproduced in an independent experiment using three Vγ9Vδ2 T cell clones from the same donor (MK) (**Supplemental Figure 3**).

These outcomes suggest that expression of HBZ and Tax proteins enhance the cytolysis of the target cells by Vγ9Vδ2 cells.

### γδ T cells expanded by Zoledronate or Concanavalin A can kill HBZ- and tax-expressing cells

**Figure 5:**
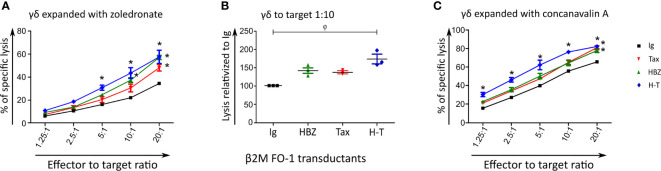
Expanded polyclonal γδ cells can kill HBZ- and H-T-expressing target cells. Cytolysis of β2M FO-1 Ig (control), HBZ, Tax or H-T transductants by expanded polyclonal γδ cells from one donor (CC) using the Cr^51^ release assay. Vγ9Vδ2 T cells were expanded with Zoledronate **(A, B)** or Concanavalin A **(C)**. For **(A, C)** difference with Ig with *: p<0.001. p-values were determined by Two-way ANOVA, with Bonferroni post-test. **(B)** Percentage of increased cytolysis of γδ cells in a dilution 1/10 effector to target, for two independent experiments using the same donor. φ: p<0.01; p-values were determined by Kruskal-Wallis test, with Dunns post-test.

Finding the most intriguing results on the transduced β2M FO-1 cells, we next aimed to determine whether the enhanced cytotoxicity of Vγ9Vδ2 clones could be reproduced with expanded human polyclonal γδ cells. To this end, polyclonal γδ cells from a healthy donor were expanded using Zoledronate (**Figures 5A, B**) or Concanavalin A (**Figure 5C**) following published protocols (27). In support to our previous results using γδ T cell clones, the cytotoxicity of human polyclonal γδ T cells was statistically higher when the target cells expressed HBZ and Tax together and a tendency of the same effect is observed when HBZ and Tax are expressed alone (**Figures 5A**, **B**). Cytotoxicity by γδ T cell expanded with Concanavalin A showed significant increase when HBZ and Tax were expressed (**Figure 5C**).

### IFN-γ release does not correlate with the cytotoxicity when HBZ and tax are expressed

Given the range of results obtained during the cytolysis experiments, we measured interferon γ (IFN-γ) levels by ELISA to corroborate the recognition of the Peer and β2M FO-1 transductants by the γδ T cells.

**Figure 6:**
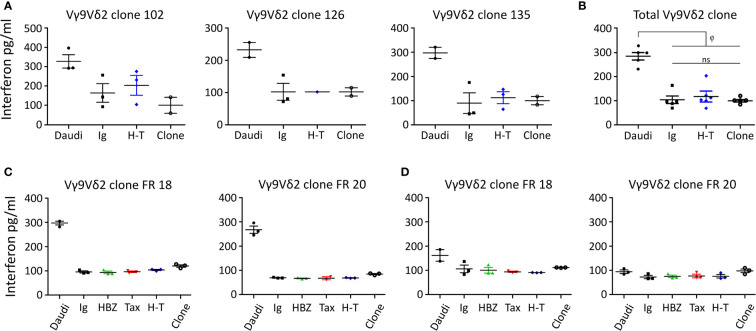
IFN-γ release by Vγ9Vδ2 clones in contact with the β2M FO-1 transduced cells. Assay made with **(A)** three Vγ9Vδ2 T clones. **(B)** pulled valuves from **(A, C, D)** IFN-γ release assay using two Vγ9Vδ2 T clones from one donor (FR) **(C)** in absence or **(D)** presence of mevastatin; φ: p<0.01. ns: not significant. p-values were determined by One-way ANOVA, with Bonferroni post-test.

**Figure 6** shows that Vγ9Vδ2 clones can release IFN-γ in contact with Daudi cells. Nonetheless, there was no significant difference in IFN-γ release between the β2M FO-1 cells transduced with H-T, control (Ig) or the clones alone (**Figure 6B**). This effect can be observed for each particular clone (**Figures 6A**, **C**, **D**) as well as for all the clones together (**Figure 6B**). On the contrary, there was an increase in cytotoxicity when Vγ9Vδ2 clones were in contact with β2M FO-1 H-T cells compared to the control (Ig) (**Supplemental Figure 4**). Hence, from those experiments, we observed no correlation between IFN-γ release and cytotoxicity.

To further understand the interaction between the Vγ9Vδ2 clones and β2M FO-1 transductants we repeated the cytolysis and IFN-γ release experiments blocking recognition by the Vγ9Vδ2 TCR. The Vγ9Vδ2 TCR binds to BTN2A1 and BTN3A1 in the presence of pAgs. One endogenous pAg is isopentenyl pyrophosphate (IPP), an intermediate in the mevalonate pathway. Mevastatin is a compound that blocks the mevalonate pathway by inhibiting HMG-CoA reductase, reducing IPP synthesis. Without IPP bound to BTN, Vγ9Vδ2 cells cannot longer recognize and kill the target cells (24).

Vγ9Vδ2 clones FR 18 and FR 20 were co-cultured with the transduced β2M FO-1 cells in the absence (**Figure 6C**) or presence of mevastatin (**Figure 6D**). Vγ9Vδ2 clones did not show differences in IFN-γ release when in contact with β2M FO-1 Ig, HBZ, Tax or H-T, compared to the same clones in the absence of target cells. Notwithstanding, Vγ9Vδ2 clones could release IFN-γ in contact with Daudi and, as expected, mevastatin reduced the production of IFN-γ.

### Tax reduces cytolysis of β2M FO-1 cells by NK clones

NK cells and γδ T cell share several membrane receptors such as NKG2D, NKp30, NKp44 and NKp46. In γδ T cells NKp30 and NKp44 have been shown to mediate granzyme B production and cytotoxicity in a TCR-independent manner (28). We hypothesized that if the cytotoxicity of NK cells and γδ T cells was similar, then the recognition and killing might be mediated by shared receptors; while if the cytotoxicity of NK cells and γδ T cells was different, the responsible receptors for the recognition and killing might differ.

**Figure 7:**
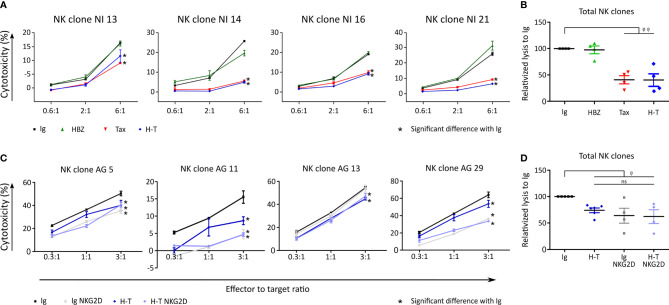
Tax expression reduces killing by NK clones. **(A)** Cytolysis of β2M FO-1 transduced cells with Ig (control, black), HBZ (green), Tax (red) and H-T (blue) by four NK clones from one donor (NI). Difference with Ig with *: p<0.001. p-values were determined by Two-way ANOVA, with Bonferroni post-test. **(B)** Normalized values from A to average Ig for each clone. φ φ: p<0.001 **(C)** Cytolysis of NK clones from one donor (AG), co-cultured with β2M FO-1 cells transduced with Ig (black) or H-T (blue) in the absence of an anti-NKG2D antibody; or Ig NKG2D (grey) or H-T NKG2D (purple) in the presence of a blocking anti- NKG2D antibody. **(D)** Normalized values from C to average Ig for each clone. φ: p<0.05 and φφ: p<0.01 represents a difference with Ig; p-values were determined by One-way ANOVA, with Dunn’s post-test.

Four different NK clones recognized and killed β2M FO-1 cells neither expressing Tax nor HBZ (**Figures 7A**, **B**). When HBZ was expressed, there was no difference to the control. When Tax was expressed, β2M FO-1 cells were partially protected from NK cytolysis.

With the aim of inhibiting the NK killing capacity, we blocked NKG2D, an activating receptor of NK cells (19). The addition of anti-NKG2D antibodies to the killing assay reduced cytolysis of the β2M FO-1 cells (**Figures 7C**, **D**).

In conclusion, Tax protects β2M FO-1 cells from NK cytolysis (**Figure 7**) but promotes cytolysis by γδ T cells (**Figures 4**, **5**). Thus, recognition of the target cells by NK and γδ T cells could differ.

### Cytolysis by Vγ9Vδ2 cells is most likely mediated through the TCR

**Figure 8:**
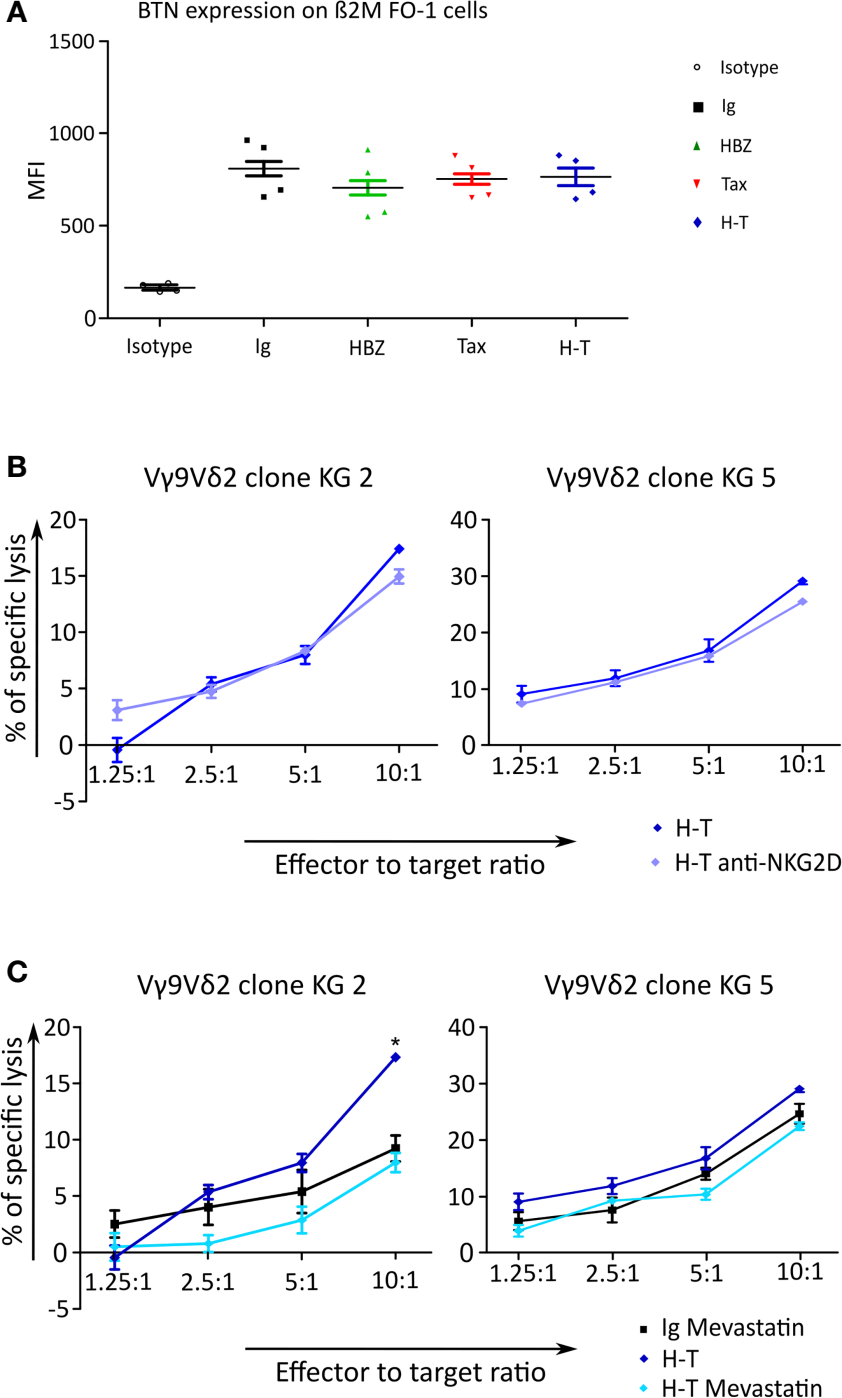
BTN expression and Cr^51^ release assay to investigate the cause of γδ cells killing response triggered by transduced β2M FO-1 cells. **(A)** Expression of BTN3A1 was measured on β2M FO-1 cells transduced with Ig, HBZ, Tax or H-T in four independet experiments by staining with an anti-BTN3A1 antibody and quantification by flow cytometry. The mean fluorescence intensity (MFI) is given. **(B, C)** Cr^51^ release assay using two Vγ9Vδ2 clones as effectors from one donor (KG) and β2M FO-1 cells transduced with H-T as target was done. **(B)** β2M FO-1 H-T cells in the absence (blue) or presence (purple) of anti-NKG2D. **(C)** β2M FO-1 Ig cells in the presence of mevastatin (black) and β2M FO-1 H-T cells in absence (blue) or presence (light blue) of mevastatin. *: p<0.01 and represents a difference with H-T; p-values were determined by Two-way ANOVA, with Bonferroni post-test.

A possible explanation for the increased killing of β2M FO-1 cells expressing HTLV-1-derived proteins by Vγ9Vδ2 cells might be an increase in the expression of BTN3A1. BTN3A1 is a crucial protein that binds pAgs and allows the recognition by the Vγ9Vδ2 TCR. To identify if the transduction with HBZ, Tax or H-T might influence the expression of BTN3A1 in β2M FO-1 cells, we stained the transductants with an anti-BTN3A1 antibody an analyzed them by flow cytometry. We could not observe differences regarding the expression of BTN3A (**Figure 8A**).

Another explanation for the increased killing might be due to NKG2D. This receptor is also present in γδ T cells and may enhance their response (29). With the aim of testing whether this would occur in our system, we included the blocking anti-NKG2D antibody in the cytotoxicity experiment using β2M FO-1 H-T cells as target and two Vγ9Vδ2 clones as effector cells. We did not observe an effect of blocking NKG2D on the cytotoxicity by Vγ9Vδ2 clones (**Figure 8B**).

Finally, in β2M FO-1 H-T cells, the cytotoxicity by Vγ9Vδ2 clones was reduced in the presence of mevastatin (**Figure 8C**), and mevastatin did not have an effect in BTN3A1 expression (**Supplemental Figure 5**). These findings suggest that the mevalonate pathway could be involved in the recognition of HBZ and Tax double-expressing cells.

## Discussion

The main goal of this study was to contribute to the knowledge of γδ T cells in HTLV-1 infection. We show that the frequency of γδ T cells in HTLV-1 patients is altered compared to NI individuals, and that γδ T cells show cytotoxic activity towards target cells expressing the HTLV-1 proteins HBZ and Tax. Of note, there was no correlation between the killing activity of γδ T cells and their IFN-γ release.

Here, we document that the γδ T cell repertoire, Vγ9Vδ2 T cells in particular, is altered in HTLV-1 infected patients by two different techniques: flow cytometry and spectratyping. Vγ9-positive γδ T cells were reduced in HTLV-1 patients and this was more pronounced in the individuals with pathologies. Considering that Vγ9 pairs in 95% of the cases with Vδ2 (30), our data showed that Vγ9Vδ2 T cells are reduced in HTLV-1 infected patients. This alteration is in line with data presented at a meeting of the International Society for Neurovirology by Sato et al. (31), and with a report that also found diminished Vγ9Vδ2 T cells in HAM/TSP patients (32). Contrarily, another publication did not report an alteration of the Vγ9Vδ2 T cells in HTLV-1 infected patients but described changes in the phenotype and function of γδ T cells (33). This discrepancy could be due to the fact that in our study, patients with autoimmune or inflammatory diseases or other co-infections were excluded as they could also alter the percentage of different T cell populations as is the case for lymphomatic malignancies (34). For example, one NI patient was excluded, since the patient was HIV positive. In this particular sample we observed an inverted Vδ2/Vδ1 ratio (δ2 = 1.2% and δ1 = 2,0%), as it has been reported for HIV-infected persons (25). Thus, one limitation of the selection method used (excluding patients with other diseases) is that our sample group is small, therefore more research would be useful to reinforce these findings.

Patients with HTLV-1 pathologies show a high PVL and this clearly correlated with a significant decrease of the Vγ9Vδ2 cells. A similar decrease was reported of other cytotoxic cells. NK cells were decreased in HAM/TSP patients and a significant inverse correlation between the PVL and the frequency of NK cells in HTLV-1-infected individuals was found (17). Tax-specific CD8+ CTLs in ATLL patients were present at lower frequencies (35). In addition, an inverse correlation between the PVL and the frequency of perforin+ CD8+ T cells has been observed in all HTLV-1 infected individuals. Thus, the CTL response plays a critical role in limiting HTLV-1 and the risk of the inflammatory disease such as HAM/TSP (36). In the same way as NK and CTL cells, we hypothesize that less γδ T cells could cause an insufficient cytotoxic response. Reduction in γδ T cells, especially the Vγ9Vδ2 subset could be driven by HTLV-1 infection directly or indirectly. Although infection of γδ T cells by HTLV-1 *in vitro* is possible (37), there is no evidence that this infection could occur *in vivo*. A possible indirect mechanism for the reduction of γδ T cells could be γδ T cell exhaustion. CTL exhaustion has been reported in HTLV-1 infected individuals (38). The γδ T cells could face the same fate, containing the virus spread until exhaustion and getting lost from the system by clonal attrition, leading to the disease progression. Such reactive changes in the periphery contrast the acquired immunodeficiency in thymoma patients, which is characterized by polyclonal, cytotoxically inert γδ T-cell populations of central origin (39, 40). Thus, modulating the viral strategy that induces a balance between promotion and limitation of infected T cell expansions could be the key to control HTLV-1 associated diseases.

In conclusion, the correlation between a high PVL and low amount of Vγ9Vδ2 T cells, especially in those patients with HTLV-1 associated pathologies, suggests that γδ T cells could control the spreading of the virus. Thus, the second part of our study focused on testing whether γδ T cells could have a role in containing the infection. To this end we generated cell lines expressing the HTLV-1 proteins HBZ and Tax, allowing us to dissect the impact of each protein individually on the activity of γδ cells.

Here we show that Vδ2Vγ9 T cell clones are able to kill β2M FO-1 cells when expressing HBZ alone and also kill Peer and β2M FO-1 cells when co-expressing HBZ and Tax. Additionally, polyclonal γδ T cells expanded with Zoledronate or Concanavalin A have the same effect on β2M FO-1 cells co-expressing HBZ and Tax. Zoledronate expands Vγ9Vδ2 T cells, while Concanavalin A expands Vδ1 and Vδ2 T cells (41). Thus, the cytotoxicity of the latter might have been due to the Vγ9Vδ2 T cells. The expanded Vδ1 T cells could be responsible for the increased cytolysis of β2M FO-1 Ig cells by Concanavalin A, compared to Zoledronate-expanded γδ T cells. The killing of Peer cells expressing Tax alone by Vγ9Vδ2 T cells was not increased compared to the control Peer cells, but the killing of Peer cells expressing HBZ and Tax simultaneously was increased. We propose that the expression of both HBZ and Tax is necessary to increase the levels of phospho-antigens by Peer cells.

We also compared the cytolysis of HBZ- and Tax-expressing target cells by NK and γδ T cells. We hypothesized that if the cytotoxicity of NK cells and γδ T cells was different, the responsible molecule for the recognition could be the TCR present only on γδ T cells. β2M FO-1 Ig control cells were killed by NK but not by γδ T cells. The expression of HBZ and Tax made them susceptible to γδ T cell killing, but expression of Tax protected them from NK cell killing, suggesting that the cytotoxicity is triggered by different mechanisms. We think that the protective role of Tax towards NK cell killing is given either by upregulation of inhibitory NK ligands or downregulation of NK cell activating ligands (42). Indeed, NKG2D receptors were involved in NK but not in γδ T cell cytotoxicity. These results concur with previous reports showing that NKG2D is one of the main receptors that leads to activation of NK cells but only as an “assistant” receptor that potentiates TCR-mediated responses in γδ T cells (29). In the case of β2M FO-1, the expression of one protein (Tax or HBZ) increased the cytotoxicity by Vγ9Vδ2 T cells, and was inhibited by mevastatin, a drug that reduces phospho-antigen levels, suggesting that these proteins act on the mevalonate pathway. Indeed, HBZ and Tax did not increase BTN3A1 expression.

Enhanced cytotoxicity by Vγ9Vδ2 T towards cells expressing HBZ and/or Tax might be triggered by dysregulation of the mevalonate pathway. This is in line with the fact that these HTLV-1 proteins alter transcription of the host cells (5–7). In accordance with our results, an inhibitor of the mevalonate pathway (namely incadronate) prevents cell growth of HTLV-1-infected T cell lines and primary ATLL cells, but not of NI T cell lines or normal PBMCs (43). Furthermore, HTLV-1 alters the lipid profiles (44), which indicates that this alteration could be caused by a deregulation in the metabolism of the infected cell. Together our data suggest that recognition by γδ T cells is triggered because HBZ and Tax dysregulate the mevalonate pathway, resulting in an increase of the phospho-antigen IPP which can be recognized by Vγ9δV2 TCR. Thus, the TCR would not recognize Tax or HBZ directly. However, we have not directly measured the phospho-antigen levels in those cells. As reported earlier, killing by Vγ9Vδ2 T cells recognizing phospho-antigens might be done by secreting perforin, granzyme B, and granulysin (26).

An unexpected result was the discrepancy between γδ T cell-driven cytotoxicity and INF-γ production, since it is generally accepted that IFN-γ is a good surrogate marker for cytotoxicity (45). However, recent evidence suggests that this might not be the case; for example, the increased IFN-γ production by γδ T-cells due to PD-1 blockade was not accompanied by an increase in specific cell dependent cytotoxicity against leukemia (46). Our data shows that an increased cytotoxicity does not necessarily correlate with an increase of IFN-γ release. One possibility is that IFN-γ it is indeed produced but cannot be released. This scenario has been already described for CD8+ tumor-infiltrating lymphocytes, since it was shown that those cells produce normal amounts of intracellular cytokines, but fail to secrete them, because of defective actin rearrangements at the synapse (47). Alternatively, IFN-γ production and cytotoxicity of γδ T cells might be regulated independently from each other.

In this study we focus on the ability of γδ T cells to recognize β2M FO-1 (myeloma cell line) and Peer (γδ T cell line) cells expressing HBZ and Tax. However, the natural target of HTLV-1 are CD4+ T cells. Thus, it would be good to repeat the experiments expressing HBZ and Tax in CD4+ T cells and testing them for the susceptibility to be killed by Vγ9Vδ2 T cells. Since β2M FO-1 and Peer cells were both better killed in the presence of HBZ and Tax, we expect to observe the same enhancement of killing in the case of CD4+ T cells.

In conclusion, our results indicate that HTLV-1 infection induces changes in the numbers of γδ T cells with a reduction of Vγ9Vδ2 T cells when the HTLV-1 pathologies, HAM/TSP and ATLL, are developed. In addition, we show that Vγ9Vδ2 T cells can recognize and kill cells expressing HBZ and Tax, opening the possibility that Vγ9Vδ2 cells contribute to HTLV-1 containment. We show that HTLV-1 associated disease progression and deregulation of γδ T cells are closely related. We also consider that enhancing an immunological equilibrium of long-term functional cytotoxic T cells, including γδ T cells, could be a therapeutic strategy for preventing the onset of HTLV-1-associated diseases. Nonetheless, a verification that HTLV-1 do not infect γδ T cells is indispensable to use these cells as therapeutic strategy. Overall, our findings provide valuable insights into the systemic immune dysregulation associated with HTLV-1 malignancies and could guide future research to use γδ T cell as effective immunotherapy for HTLV-1 related pathologies.

## Materials and methods

### Subjects

This study enrolled 2 ATLL, 5 HAM/TSP patients and 16 HTLV-1 AC and 21 NI individuals, which were included as controls (**Supplemental Tables 1**, **2**) attending the Instituto de Investigaciones Biomédicas en Retrovirus y SIDA (INBIRS) UBA – CONICET, Argentina. Samples from the same HTLV‐1‐infected patient were taken on different days. The diagnosis of ATLL and HAM/TSP was based on the World Health Organization criteria. The study protocol was approved by the Institutional Review Board as well as by the External Ethical Committee (NEXO AC IRB#0005349, protocol #1563). A written informed consent was obtained from all individuals before blood collection. In addition, patients were interviewed, and epidemiological and clinical data was obtained. Patients with autoimmune or inflammatory diseases or other co-infections were excluded from the study.

### HTLV-1 confirmation

Plasma samples were screened for the presence of anti-HTLV-1/2 antibodies (ELISA HTLV IandII Ab, ULTRA version, Diapro). For molecular confirmation, DNA was extracted from peripheral blood mononuclear cells (PBMCs) by column extraction (ADN PuriPrep-S kit, Highway, Inbio) and analyzed by nested polymerase chain reaction (n-PCR) for HTLV-1/2 following W Heneine et al. protocol (48).

### Proviral load quantification

DNA was extracted from peripheral blood mononuclear cells (PBMCs) by column extraction (ADN PuriPrep-S kit, Highway^®^, Inbio, Tandil, Argentina). Absolute quantitation of PVL was performed by real-time SYBR Green PCR, using an ABI Prism 7500Prism System (Applied Biosystems, Foster City, CA, USA) as previously described (49).

### PBMCs isolation and flow cytometry analysis

Peripheral blood mononuclear cells (PBMCs) were isolated from peripheral blood by standard Ficoll-Hypaque (GE Healthcare) density gradient centrifugation. Dead cells were excluded using fixable viability dye Fixable Near-IR Dead Cell Stain Kit (Invitrogen; dilution 1:10). For surface marker staining, cells were incubated with antibodies at 4°C for 30 min and then washed and fixed. The following fluorescence antibodies were applied: anti-TCR Vγ9 (BioLegend; clone B3), anti-CR Vδ2 (BioLegend; clone B6), anti-TCRγδ (BD; clone B1), anti-TCR Vδ1, human (Miltenyi; clone REA 173), and anti-CD3 (Biolegend, clone UCHT1). All samples were acquired with a BD FACS CANTO A (BD Biosciences) and analyzed with FlowJo software.

### Subject’s RNA isolation and cDNA synthesis

Total RNA was isolated from PBMCs by using TRIzol (Invitrogen). All samples (**Supplemental Table 2**) were treated with RQ1 RNase-Free DNase (Promega) and 1 μg of total RNA was submitted to RT-PCR in order to obtain the complementary DNA by using a MMLV (Promega) according to the manufacturer׳s instructions. The cDNA was quantified using a Nanodrop 2000C (Thermo Scientific) and stored at -20°C.

### Spectratyping

The samples included belong to 7 non-infected (NI) (n=7), 10 AC (n=11), 4 HAM/TSP (n=5) and 2 ATLL individuals (n=3) (**Supplemental Table 2**). Primer sequences and procedures can be found in Christopoulos P. et al. (20).

### Plasmids and sequence

Tax and HBZ sequences and pMXs-IRES-GFP (Ig) plasmid were donated by Junichiro Yasunaga, M.D., Ph.D. (Laboratory of Virus Control, Institute for Virus Research, Kyoto University, Japan). Ig retroviral expression vector was used together with the Platinum-GP cell line to generate viral particles, which were later used to deliver the genetic material into Peer and β2mFO-1 cells. The cloning of Tax and HBZ into Ig was done using the restriction enzymes BamH1, XhoI and EcoR1.

### Cell culture

#### Cell lines

Cell lines K562, Raji, Peer and β2mFO-1 were cultured in RMPI 1640, supplemented with 2 MM L-glutamine, 100 IU/ml penicillin, 100 Ag/ml streptomycin (all Thermo Fisher), and 10% (or 20% for Peer) of fetal bovine serum (FBS) (HyClone). Daudi cells were maintained in IMEM 2 MM L-glutamine, 100 IU/ml penicillin, 100 Ag/ml streptomycin (all Thermo Fisher) containing 10% FBS (HyClone). Platinum-GP (Plat-GP) retroviral packaging cell line (Cell Biolabs, Inc.) were maintained in DMEM 2 MM L-glutamine, 100 IU/ml penicillin, 100 Ag/ml streptomycin (all Thermo Fisher) containing 10% FBS (HyClone). Mycoplasma contamination was excluded.

#### Virus production

Virus production was accomplished following LipofectamineTM3000 Reagent (ThermoFisher) protocol instruction. Plating 1*106 Plat GP cells into a six well plate and adding 250 µl of Opti-MEM (ThermoFisher); 3.75 µl lipofectamin reagent; 2.5 µg of plasmid and 10 µl of P3000 reagent. After two days, the medium was collected and used to infect the target cell lines. Infection was confirmed by GFP expression and or RT-PCR.

### RT-PCR to confirm tax and HBZ presence

#### Cell line RNA isolation and cDNA synthesis

Total RNA was isolated by using column RNA kit (Qiagen) and RNase A DNase and protease-free (ThermoFisher). Af-ter extraction, 1 μg of total RNA was used for RT-PCR by using First-strand cDNA Synthesis Kit (GE Healthcare) ac-cording to the manufacturer׳s instructions. The cDNA was quantified using a Nanodrop 2000C (Thermo Scientific) and stored at -20°C.

#### PCR amplification

The PCR amplification was performed in a 25 μl reaction volume containing PCR Buffer 1x, Q solution 1x, MgCl2 2,5 mM, dNTPs 0,2 mM, Primers 0,3 mM, Taq polymerase 0,5 units (Qiagen, Hilden, Germany) and 2 μl of cDNA (50 ng/μl). Primers were chosen based on their location within the prototype HTLV-1 genomic sequence available in the GenBank J02029.1, considering conserved sequences, and designed to avoid complementary sequences and strong internal sec-ondary structures. Tax primers Fw (5´ CCGTTGTCTGCATGTACCTCTAC 3´) and Rv (5´ CGTTTTGCCAGGCTGTTAG 3´). HBZ primers Fw (5´ CGCGGATCCCGCGGATCCCGATTATG 3´) and Rv (5´ CTTCTCCTCAGCCCGTCGCTGC 3´) were selected.

#### Western blot

5 × 106 cells were lysed in 100 µl lysis buffer containing 20 mM Tris-HCl pH8, 137 mM NaCl, 2 mM EDTA, 10% glycerol, 1x protease inhibitor cocktail, 1 mM PMSF, 5 mM iodoacetamide, 0.5 mM sodium orthovanadate, 1 mM NaF, and 0.5% Brij96 for 30 min at 4 °C followed by 15 min centrifugation to pellet the nuclei and insoluble material. The supernatants (cell lysates) were separated by 12% reducing SDS-PAGE. The separated proteins were transferred to PVDF membranes by semi-dry transfer. After blocking with 5% milk in PBS containing 0.1% Tween-20 the membranes were incubated with antibodies against HBZ (1:2000) provided by Greta Forlani (50), Tax (1:1000) (Covalab, clone 1A3), β-Actin (1:1000) (Santa Cruz, clone C4) in PBS with Tween followed by incubation with HRPO-conjugated secondary antibodies (1:10000). Western blot signals were recorded using an Image Quant LAS 4000 Mini from GE Healthcare Life Sciences.

### γδ and NK clones

PBMCs were isolated by Ficoll-Hypaque sedimentation of heparinized blood samples from healthy donors attending the Uniklink Freiburg. Cells were positively selected by FACS Aria Fusion using anti-γδ antibody (BD; clone B1, 1:100) for γδ cells and antibody anti-CD56 Pacific Blue (Biolegend 1:200) and anti-CD3 PE (Beckman Coulter 1:200) (negative selec-tion) for NK cells. The sorted cells were cloned by limiting dilution at one cell per well. The culture medium was DMEM, supplemented with 2 MM L-glutamine, 100 IU/ml penicillin, 100 Ag/ml streptomycin (all Thermo Fisher), and 10% heat inactivated human serum (PAN Biotech).

The plated cells were stimulated with 500 U/ml IL-2 (Proleukin, Novartis), 0.25 μg/ml PHA (Remel Europe Ltd.) and a mixture of irradiated allogeneic or autologous feeder cells (2x104 PBMC and 1x104 721 or 221 cell line) as described by R J van de Griend et al. (51, 52). The clones were expanded by re-plating at weekly intervals with feeder cells, fresh medium, IL-2 and PHA.

### γδ cells expanded with Zoledronate or Concanavalin A

Human peripheral blood mononuclear cells (PBMCs) were isolated from one healthy donor on a Ficoll-Hypague gradi-ent using a density centrifugation. Cells were adjusted to 106 cells/ml in RPMI 1640 medium supplemented with 10% Fetal Bovine Serum (FBS), 10 mM HEPES buffer solution, 100 U/ml % penicillin/streptomycin, 1 mM sodium pyruvat, Non-essential AS (1X).

For the expansion of Vγ9Vδ2T cells were stimulated with recombinant IL-2 (rIL-2) (50 IU/ml) and Zoledronate (2.5 μM). The purity of expanded primary human γδ T cells was analyzed by flow cytometry on day 12 in culture and the purity was > 96%.

For the expansion of Vδ1 and Vδ2 T cells, PBMCs were stimulated with Concanavalin A (1 μg/ml), rIL-2 (100 IU/ml) and IL-4 (10 ng/ml). To separate γδ T cells from T cells a negative magnetic cell separation was performed on day 14 in cul-ture using the TCR γδ T Cell Isolation Kit from Milenty Biotec. The purity of expanded primary human γδ T cells was analyzed by flow cytometry on day 20 in culture and purity was > 95%.

### Cell line and clone’s phenotypic characterization

For direct and indirect fluorescence, cells were incubated with specific antibodies diluted in PBS 1% FBS at 4°C for 30 min. Cells were washed then, if necessary, a second antibody was used. In each experiment, nonspecific binding was subtracted using appropriate isotype controls. Cells were analyzed by flow cytometry (BD LSR II, BD Biosciences). The data was analyzed by Flow Jo software (Tree Star).

Vγ9Vδ2 clones were confirmed using APC anti-TCR Vγ9 (BioLegend; clone B3 1:200) and PE anti-TCR Vδ2 (BioLegend; clone B6 1:200) antibodies. Vγ9Vδ2 and NK clones were tested against Daudi, K562 and/or Raji to determine their cyto-toxic response.

BTN3A1 was measured using anti-BTN antibody BT3-103.2 (10 μg/mL) provided by Daniel Olive, M.D., Ph.D. (Institute Paoli Calmettes) and BV421 anti-mouse IgG secondary antibody (BioLegend; clone Goat Polyclonal IgG, 1:200).

### Cell-mediated lysis assay

γδ and NK clones were used as effector cells in chromium release assays as previously described in Fisch et al., (53). Briefly, clones were serially diluted in round bottom 96-well plates (Falcon) to define E:T ratios in triplicates. Then, 5,000 targets/well, labeled for 1 h with 250 μCi/well Na251CrO4 (PerkinElmer) were used. Following 4 or 6 hs incubation (37°C, 7% CO2), the plates were centrifuged and the supernatant (50 μl) was transferred to Luma plates (PerkinElmer), which were air-dried and measured on a TopCount microplate scintillation counter (PerkinElmer). Percent specific lysis was calculated as 100 × [(counts per minute released with effectors − counts per minute released alone)/(counts per minute released by detergent) − (counts per minute released alone)]. Anti-Butyirophiylin antibody BT3-103.2 (10 μg/mL) provided by Daniel Olive, (Institute Paoli Calmettes)(see “Phenotypic characterization”) was used to inhibit butyrophil-in activation. Anti-NKG2D antibody (Beckman Coulter, clone ON72, 1:100) was used to block NKG2D activation. Mevastatin (5 μM) was used to inhibit the mevalonate pathway.

### IFN release assay

The concentrations of IFNγ were measured by standard enzyme linked immunosorbent assay (ELISA) (BD Biosciences) using the 24 hs culture supernatants of 30,000 effector 50,000 target cells. The readout was done using a Tecan Spark plate reader.

### Spectratype statistical analysis

Numerical data were analyzed with the Student’s t-test, and for correlation between numerical and categorical data, the Spearman’s coefficient was computed. The comparison of spectratyping results (i.e. area under each curve as a proxy for the abundance of the respective γδ T-cell subset, number of spectratyping peaks as a proxy for the diversity of the re-spective γδ T-cell subset, and number of clonal expansions as a proxy for the number of reactive clones) were compared across volunteer groups with the NI group as a reference, using a mixed linear model with volunteer group as a fixed effect, and Vγδ family as a random effect, in SPSS version 24 (IBM, Armonk, NY, USA). For each spectratype, a clonal expansion was defined as a peak with an area larger than the average area + 2 standard deviations of all other peaks in the same spectratype.

## Data availability statement

Publicly available datasets were analyzed in this study. This data can be found here: https://www.ebi.ac.uk/ena/browser/view/MN453119 https://www.ebi.ac.uk/ena/browser/view/AB038239.

## Ethics statement

The studies involving human participants were reviewed and approved by University of Buenos Aires, Institutional Review Board and the External Ethical Committee. The patients/participants provided their written informed consent to participate in this study.

## Author contributions

MR and WS contributed to conception and design of the study. MR, ND and CB organized the database. MR, ND, VP and PC performed the statistical analysis. MR, VP and CJ carried out the investigation. MR, MQ and WS analyzed the results. MR, ND, VP and CB obtained and prepared the sample material. MR and ND prepared the figures. MR wrote the first draft of the manuscript. MQ, PC and WWS wrote sections of the manuscript. SM, MB, MQ, PC and WS contributed to manuscript revision. All authors read and approved the submitted version.

## Funding

This work was funded by the International Cooperation Project MINCYT-BMBF 2014 (Bilateral project Argentina-Germany AL/14/08-01DN15025) (to MB), National Scientific and Technical Research Council (CONICET: PICT 20161033) (to MB), German Academic Exchange Service (DAAD) (to MR), the Deutsche Forschungsgemeinschaft (DFG, German Research Foundation) through EXC-294 (Center for Biological Signalling Studies, BIOSS), EXC-2189 (Center for Integrative Biological signalling Studies, CIBSS), SCHA976/7-1, SFB 1381 (Project A09), SFB1479 (Project ID: 441891347 - P15) and FOR2799 (MI1942/3-1 and SCHA976/8-1). The funders had no role in the study design, the collection, analysis and interpretation of the data, nor in the decision to publish, or preparation of the manuscript.

## Acknowledgments

All this work was initiated by Prof. Paul Fisch (1959-2018) and we dedicate this publication to him. We thank María Agostina Di Renzo for discussions and reading of the manuscript, and Klemens Fröhlich for productive discussions. We also thank the patient assistance service team Mirta Villa, Ricardo Casime Fernando Montesano and Sergio Mazzini from the Instituto de Investigaciones Biomédicas en Retrovirus y SIDA (INBIRS), UBA-CONICET and the Lighthouse Core Facility of the Freiburg University Medical Center and the Signaling Factory of the Signaling Campus of the University Freiburg for their help. We also thank the FOR2799 “Receiving and Translating Signals *via* the Gamma Delta T Cell Receptor”. We would also like to thank the patients´ assistance team.

## Conflict of interest

The authors declare that the research was conducted in the absence of any commercial or financial relationships that could be construed as a potential conflict of interest.

## Publisher’s note

All claims expressed in this article are solely those of the authors and do not necessarily represent those of their affiliated organizations, or those of the publisher, the editors and the reviewers. Any product that may be evaluated in this article, or claim that may be made by its manufacturer, is not guaranteed or endorsed by the publisher.
