# Supplementary material for: Phenotypic and functional analysis of γδ T cells in the pathogenesis of human T-cell lymphotropic virus type 1 infection
Source: Front Immunol. 2022 Aug 11;13:920888. doi: 10.3389/fimmu.2022.920888 (PMC9403740; doi:10.3389/fimmu.2022.920888)
Supplement: Supplementary file 1 [file DataSheet_1.docx]

**Supplemental Information**

**Supplemental Table 1:** List of samples used for flow cytometry analysis. NI samples, HTLV-1 asymptomatic carriers, HAM/TSP and ATLL patients attending the Instituto de Investigaciones Biomédicas en Retrovirus y SIDA (INBIRS) UBA – CONICET, Argentina. Patients were interviewed, and epidemiological and clinical data were obtained. PVL: proviral load. ND: not determined.

| Sample Number | Gender | Number | Age | PVL | HTLV-1 Status |
| --- | --- | --- | --- | --- | --- |
| 1 | Male | 29124 | 49 | ND | NI |
| 2 | Female | 330809 | 27 | ND |  |
| 3 | Female | 331092 | 24 | ND |  |
| 4 | Female | 330898 | 46 | ND |  |
| 5 | Female | 29386 | 67 | ND |  |
| 6 | Female | 332299 | 55 | ND |  |
| 7 | Male | 329588 | 53 | ND |  |
| 8 | Female | 332361 | 48 | ND |  |
| 9 | Female | 327792 | 43 | ND |  |
| 10 | Female | 333441 | 44 | ND |  |
| 11 | Male | 333575 | 55 | ND |  |
| 12 | Male | 333570 | 53 | ND |  |
| 13 | Female | 333621 | 33 | ND |  |
| 14 | Female | 334371 | 56 | ND |  |
| 15 | Male | 331268 | 45 | ND | Asymptomatic |
| 16 | Male | 332366 | 65 | 1.75 |  |
| 17 | Female | 331066 | 47 | 0.05 |  |
| 18 | Male | 335103 | 72 | 0.08 |  |
| 19 | Female | 335186 | 56 | ND |  |
| 20 | Female | 332365 | 43 | 11.82 |  |
| 21 | Female | 29343 | 57 | 12.9 | HAM/TSP |
| 22 | Female | 33677 | 67 | 51.6 |  |
| 23 | Male | 335126 | 48 | 7.3 |  |
| 24 | Male | 332294 | 24 | 21.3 | ATLL |

**Supplemental Table 2:** List of samples used for spectratyping analysis. NI individuals, HTLV-1 asymptomatic carriers, HAM/TSP and ATLL patients attending the Instituto de Investigaciones Biomédicas en Retrovirus y SIDA (INBIRS) UBA – CONICET, Argentina. Patients were interviewed, and epidemiological and clinical data were obtained. PVL: proviral load. ND: not determined.

| Sample Number | Gender | Number | Age | PVL | HTLV-1 Status |
| --- | --- | --- | --- | --- | --- |
| 1 | Female | 2013038 | 52 | ND | NI |
| 2 | Male | 2013042 | 44 | ND |  |
| 3 | Female | 2013043 | 32 | ND |  |
| 4 | Female | 2015006 | 41 | ND |  |
| 5 | Female | 2015023 | 34 | ND |  |
| 6 | Female | 2015115 | 22 | ND |  |
| 7 | Male | 2015116 | 29 | ND |  |
| 8 | Female | 199357 | 20 | ND | Asymptomatic |
| 9 | Female | 2014039 | 38 | 3.94 |  |
| 10 | Female | 2014062 | 46 | 0.25 |  |
| 11 | Male | 2014101 | 27 | ND |  |
| 12 | Male | 2014102 | 63 | ND |  |
| 13 | Male | 2014119 | 23 | ND |  |
| 14 | Female | 2014142 | 25 | 0.75 |  |
| 15 | Male | 2014150 | 34 | 4.61 |  |
| 16 | Male | 2015026 | 70 | 0.12 |  |
| 17 | Male | 2015065 | 48 | 4.24 |  |
| 18 | Male | 2015121 | 48 | 6.55 |  |
| 22 | Female | 2014057 | 42 | 13.08 | HAM/TSP |
| 23 | Female | 2014151 | 61 | 12.86 |  |
| 24 | Female | 2015007 | 43 | 15.87 |  |
| 25 | Female | 2015064 | 43 | 17.3 |  |
| 26 | Female | 2015007 | 43 | 15.87 |  |
| 19 | Female | 2014059 | 61 | 5.03 | ATLL |
| 20 | Male | 2015078 | 18 | 9.54 |  |
| 21 | Male | 2015107 | 18 | 9.08 |  |

**Supplemental Table 3:** Computational analysis of the spectratyping: subset quantification, number of peaks and clonal expansions. p value (p-V) was calculated using Spearman’s coefficient between NI and Asymptomatic, HAM/TSP or ATLL. ns = non significant

γδ subset quantification (area)

|  | **Vδ1** | p-V | **Vδ2** | p-V | **Vδ3** | p-V | **Vδ4** | p-V | **Vδ5** | p-V | **Vδ6** | p-V |
| --- | --- | --- | --- | --- | --- | --- | --- | --- | --- | --- | --- | --- |
| NI | 3534 | ns | 32040 |  | 8344 |  | 5874 |  | 13699 |  | 1211 |  |
| ASYMPTOMATIC | 1903 | ns | 13988 | **0.003** | 2042 | **0.006** | 1505 | **0.047** | 513 | **0.001** | 24 | ns |
| HAM/TSP | 493 | ns | 8348 | **0.005** | 575 | **0.015** | 1789 | ns | 1642 | **0.024** | 1574 | ns |
| ATLL | 0 | ns | 8816 | **0.011** | 0 | **0.017** | 8010 | ns | 494 | **0.025** | 1272 | ns |

|  | **Vγ1** | p-V | **Vγ2** | p-V | | **Vγ3** | | p-V | | **Vγ4** | | p-V | | **Vγ5** | | p-V | | **Vγ8** | | p-V | | **Vγ9** | | p-V | |
| --- | --- | --- | --- | --- | --- | --- | --- | --- | --- | --- | --- | --- | --- | --- | --- | --- | --- | --- | --- | --- | --- | --- | --- | --- | --- |
| NI | 11880 |  | 29199 |  | 21243 | |  | | 12684 | |  | | 14953 | |  | | 20708 | |  | | 10990 | |  | |  |
| ASYMPTOMATIC | 2516 | **0.006** | 13442 | ns | 6219 | | **0.012** | | 4453 | | **0.042** | | 4946 | | **0.002** | | 6987 | | **0.008** | | 1446 | | **0.007** | |  |
| HAM/TSP | 629 | **0.018** | 19832 | ns | 5864 | | ns | | 3190 | | ns | | 7837 | | ns | | 8033 | | ns | | 1359 | | 0.052 | |  |
| ATLL | 4449 | ns | 15572 | ns | 10316 | | ns | | 256 | | **0.045** | | 4820 | | **0.038** | | 9389 | | ns | | 1137 | | 0.069 | |  |

|  | **Vγδ** | p-V | **Vγ9δ2** | p-V | **non Vγ9Vδ2** | p-V |
| --- | --- | --- | --- | --- | --- | --- |
| NI | 7323 |  | 13562 |  | 6425 |  |
| ASYMPTOMATIC | 1873 | **<0.001** | 2979 | **0,002** | 1671 | **<0.001** |
| HAM/TSP | 2045 | **<0.001** | 673 | **<0.001** | 2083 | **<0.001** |
| ATLL | 2642 | **<0.001** | 2075 | **0,03** | 2583 | **0,002** |

γδ number of peaks (diversity)

|  | **Vδ1** | p-V | **Vδ2** | p-V | **Vδ3** | p-V | **Vδ4** | p-V | **Vδ5** | p-V | **Vδ6** | p-V |
| --- | --- | --- | --- | --- | --- | --- | --- | --- | --- | --- | --- | --- |
| NI | 3.429 |  | 10.43 |  | 5.429 |  | 4.714 |  | 6.571 |  | 1.571 |  |
| ASYMPTOMATIC | 2.211 | ns | 6.316 | ns | 1.632 | **0.008** | 0.947 | **0.004** | 0.842 | **0.003** | 0.053 | ns |
| HAM/TSP | 1.25 | ns | 3.75 | **0.023** | 2 | ns | 2.25 | ns | 3 | ns | 1.5 | ns |
| ATLL | 0 | ns | 6.333 | ns | 0 | **0.015** | 4.667 | ns | 3.333 | ns | 2 | ns |

|  | **Vγ1** | p-V | **Vγ2** | p-V * | **Vγ3** | p-V * | **Vγ4** | p-V | **Vγ5** | p-V | **Vγ8** | p-V | **Vγ9** | p-V |
| --- | --- | --- | --- | --- | --- | --- | --- | --- | --- | --- | --- | --- | --- | --- |
| NI | 7.857 |  | 10,71 |  | 7.571 |  | 7.429 |  | 8,286 |  | 7,571 |  | 7,143 |  |
| ASYMPTOMATIC | 3.053 | **0.007** | 6,263 | **0,013** | 2.684 | **0,02** | 2.872 | **0.005** | 5,053 | ns | 5,31 | ns | 1,158 | **0,001** |
| HAM/TSP | 0.75 | **0.005** | 4,25 | **0,011** | 4.25 | ns | 3.26 | ns | 5,25 | ns | 5,705 | ns | 2 | **0,029** |
| ATLL | 2.667 | ns | 6,667 | ns | 6.333 | ns | 3.41 | **0.005** | 5,667 | ns | 5,864 | ns | 3,333 | ns |

|  | **Vγδ** | p-V | **Vγ9δ2** | p-V | **non Vγ9Vδ2** | p-V |
| --- | --- | --- | --- | --- | --- | --- |
| NI | 6,217 |  | 8.8 |  | 5.829 |  |
| ASYMPTOMATIC | 2.301 | **<0.001** | 3.759 | **<0.001** | 2.161 | **<0.001** |
| HAM/TSP | 2.676 | **<0.001** | 1.156 | **<0.001** | 2.857 | **<0.001** |
| ATLL | 2.915 | **<0.001** | 4.846 | ns | 2.683 | **<0.001** |

γδ number of clonal expansions

|  | **Vδ1** | p-V | **Vδ2** | p-V | **Vδ3** | p-V | **Vδ4** | p-V | **Vδ5** | p-V | **Vδ6** | p-V |
| --- | --- | --- | --- | --- | --- | --- | --- | --- | --- | --- | --- | --- |
| NI | 0.29 |  | 0.857 |  | 0.714 |  | 0.714 |  | 0.429 |  | 0.429 |  |
| ASYMPTOMATIC | 0.263 | ns | 0.632 | ns | 0.316 | ns | 0.263 | ns | 0.211 | ns | 0.053 | ns |
| HAM/TSP | 0.25 | ns | 0.5 | ns | 0.25 | ns | 0.5 | ns | 0.25 | ns | 0.25 | ns |
| ATLL | 0 | ns | 0.667 | ns | 0 | ns | 0.667 | ns | 0.333 | ns | 0.333 | ns |

|  | **Vγ1** | p-V | **Vγ2** | p-V | **Vγ3** | p-V | **Vγ4** | p-V | **Vγ5** | p-V | **Vγ8** | p-V | **Vγ9** | p-V |
| --- | --- | --- | --- | --- | --- | --- | --- | --- | --- | --- | --- | --- | --- | --- |
| NI | 1 |  | 0.714 |  | 0.857 |  | 0.571 |  | 0.857 |  | 0.714 |  | 0.714 |  |
| ASYMPTOMATIC | 0.526 | **0.048** | 0.684 | ns | 0.474 | ns | 0.421 | ns | 0.579 | ns | 0.474 | ns | 0.157 | **0.015** |
| HAM/TSP | 0.25 | **0.028** | 0.5 | ns | 0.5 | ns | 0.5 | ns | 0.75 | ns | 0.5 | ns | 0.25 | ns |
| ATLL | 0.333 | ns | 1 | ns | 0.667 | ns | 0.333 | ns | 0.333 | ns | 0.333 | ns | 0.333 | ns |

|  | **Vγδ** | p-V | **Vγ9δ2** | p-V | **non Vγ9Vδ2** | p-V |
| --- | --- | --- | --- | --- | --- | --- |
| NI | 0.674 |  | 0.793 |  | 0.655 |  |
| ASYMPTOMATIC | 0.379 | **<0.001** | 0.419 | **0.016** | 0.38 | **<0.001** |
| HAM/TSP | 0.393 | **0.002** | 0.338 | **0.02** | 0.414 | **0.019** |
| ATLL | 0.389 | **0.004** | 0.517 | ns | 0.371 | **0.01** |


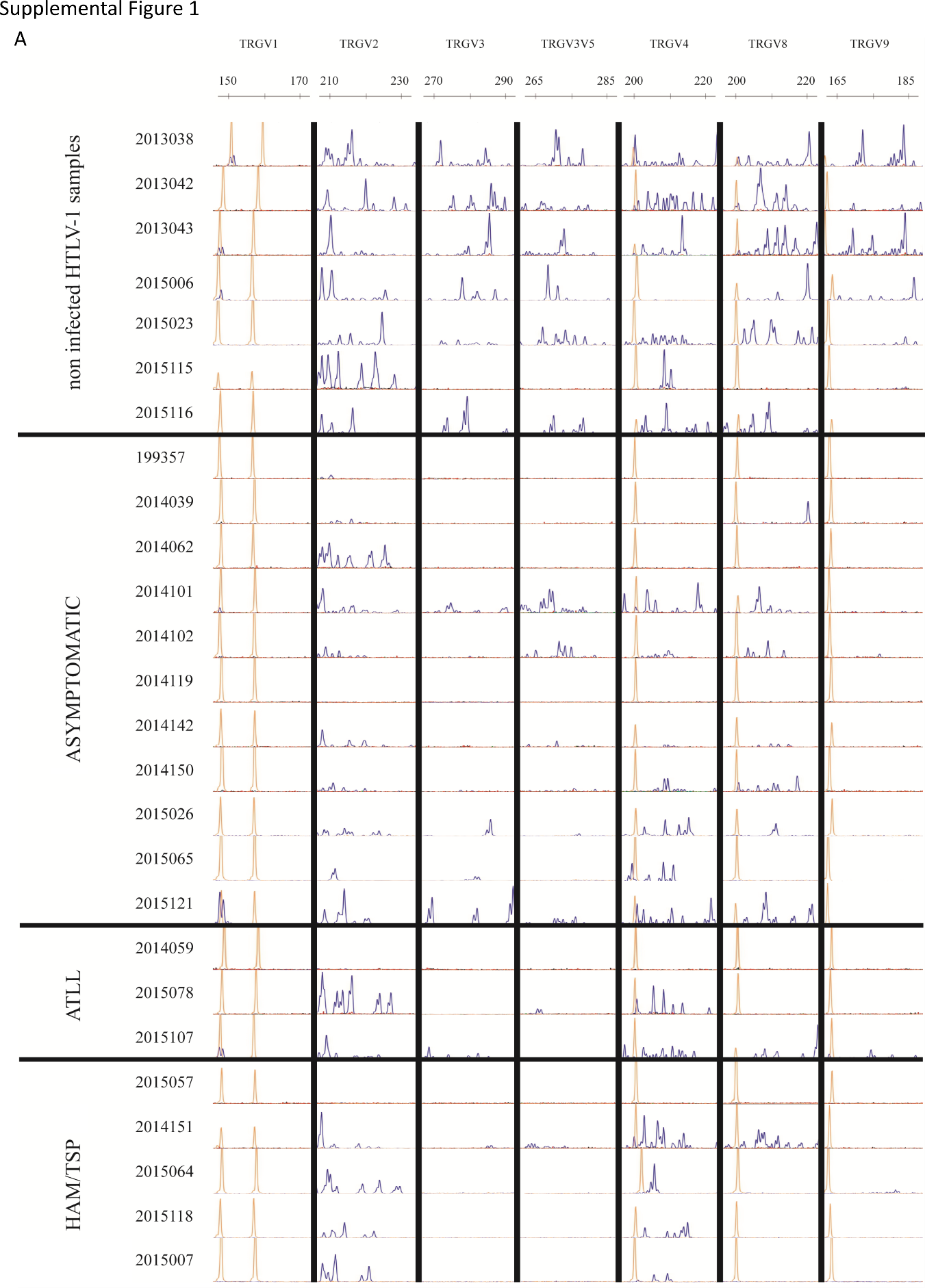

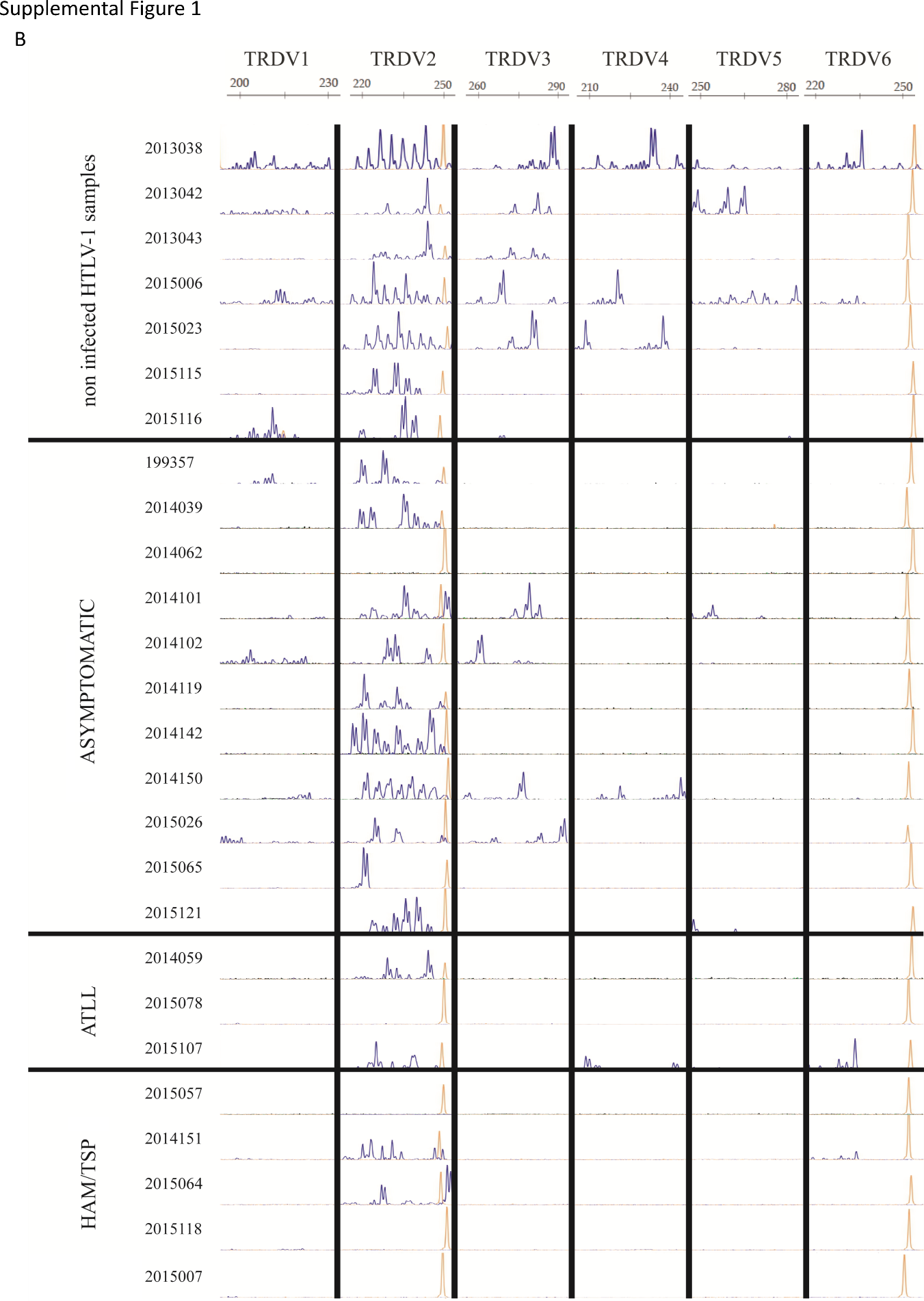


**Supplemental Figure 1:** Vγ and Vδ expression and junctional diversity. Spectratyping of **(A)** TCRγ and **(B)** TCRδ were performed with cDNA from PBMCs of 7 NI samples, 12 asymptomatics and 7 samples with patologies (4 HAM/TSP and 3 ATLL). The orange peaks represent molecular weight standards. The particular V regions amplified were the VγI subgroup (genes Vγ1-8) (pan*TRGVI*) and its individual functional genes Vγ3 (*TRGV3*), Vγ4 (*TRGV5*), Vγ5 (*TRGV3V5*), Vγ8 (*TRGV8*) and the single gene of the VγII subgroup Vγ9 (*TRVG9*), as well as Vδ1-6 (*TRDV1-6*).


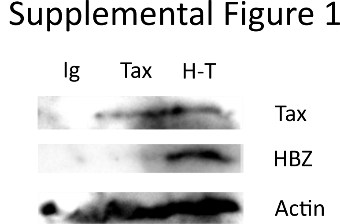


**Supplemental Figure 2:** Western blot of the Peer transductants to analyse the protein expression levels of HBZ and Tax. Actin was used as a loading control.

**
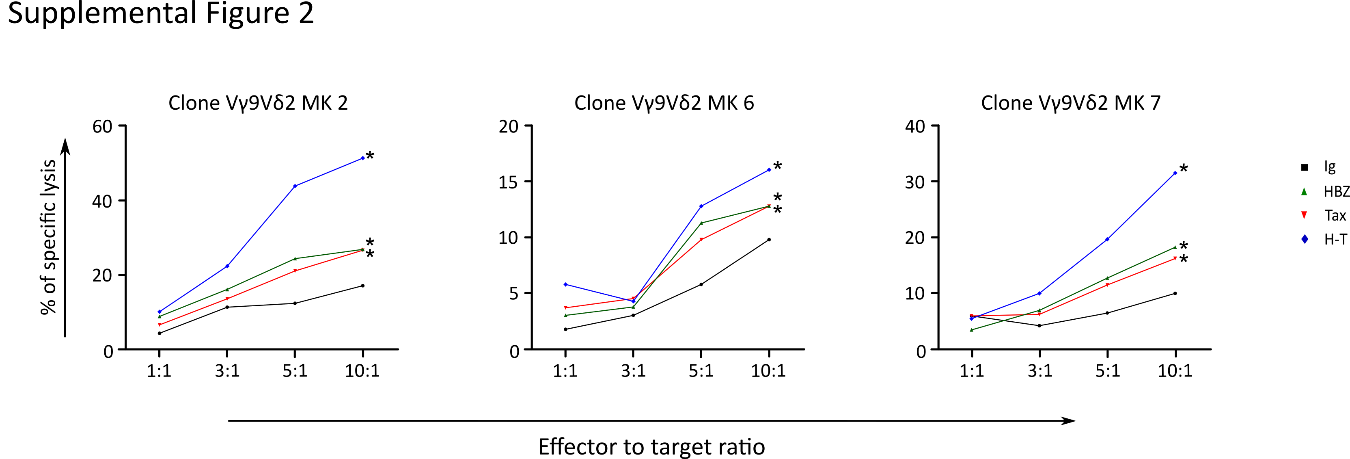
**

**Supplemental Figure 3:** Cr^51^ release assay using Vγ9Vδ2 as effectors. Cytolysis of three Vγ9Vδ2 T clones from one donor (MK), co-cultures with β2M FO-1 cells. Difference with Ig with *: p<0.001. p-values were determined by Two-way ANOVA, with Bonferroni post-test.

**
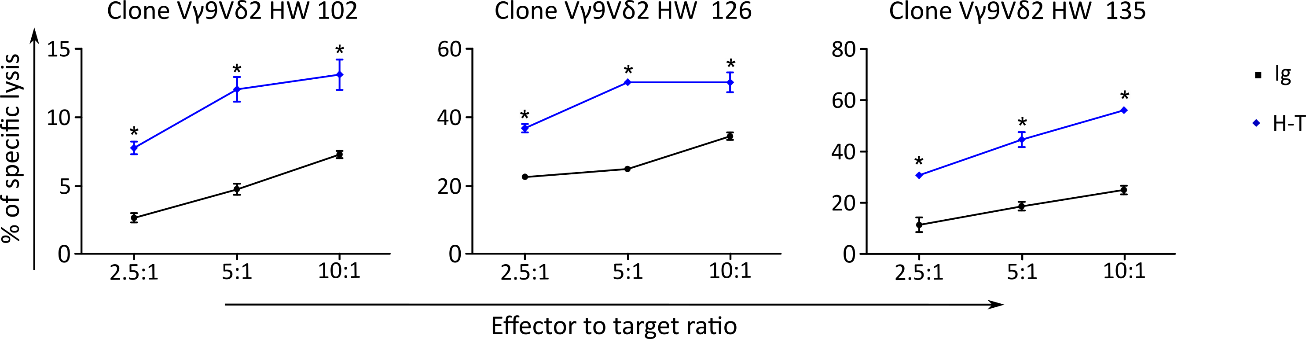
**

**Supplemental Figure 4:** Cr^51^ release assay using Vγ9Vδ2 clones as effectors. Cytolysis of three Vγ9Vδ2 T clones from one donor (HW), co-cultures with β2M FO-1 cells transfected with Ig (black) or H-T (blue). p-values were determined by Two-way ANOVA, with Bonferroni post-test; *: p<0.001.


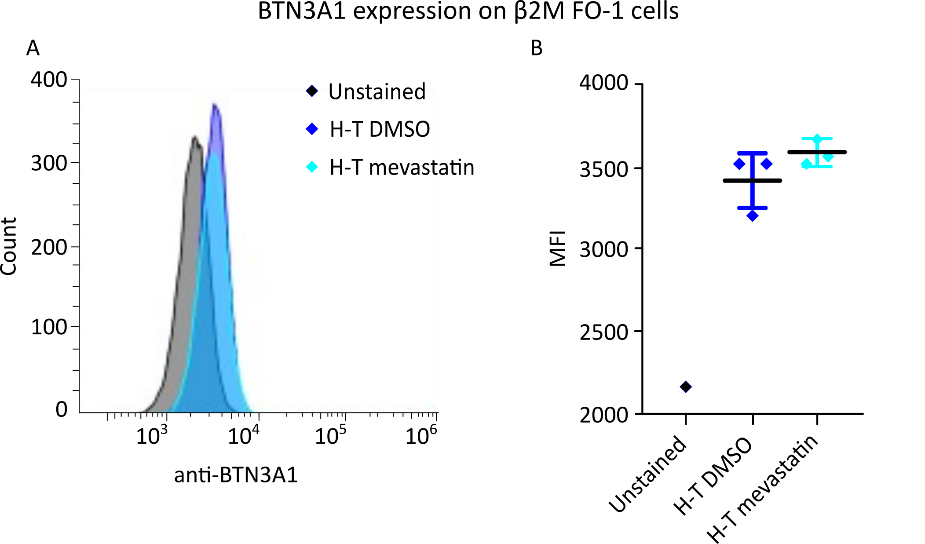


**Supplemental Figure 5:** Flow cytometry analysis of BTN3A1 surface expression on β2M FO-1 cells in absence (DMSO) and presence of mevastatin. A) Histogram representation of one replicate from one experiment B) The mean fluorescence intensity (MFI) of three replicates from one experiment is shown. The experiment was repeated two times and no significant differences were observed.
